# How to arrange the robotic environment? Leveraging experience in both motion planning and environment optimization

**DOI:** 10.3389/frobt.2024.1468385

**Published:** 2024-11-15

**Authors:** Jiaxi Lu, Ryota Takamido, Yusheng Wang, Jun Ota

**Affiliations:** ^1^ Department of Precision Engineering, School of Engineering, The University of Tokyo, Tokyo, Japan; ^2^ Research into Artifacts, Center for Engineering (RACE), School of Engineering, The University of Tokyo, Tokyo, Japan

**Keywords:** industrial robotics, motion planning, environmental arrangement, takt time, optimization, hierarchical algorithm, experience reuse, intelligent manufacturing

## Abstract

This study presents an experience-based hierarchical-structure optimization algorithm to address the robotic system environment design problem, which combines motion planning and environment arrangement problems together. The motion planning problem, which could be defined as a multiple-degree-of-freedom (m-DOF) problem, together with the environment arrangement problem, which could be defined as a free DOF problem, is a high-dimensional optimization problem. Therefore, the hierarchical structure was established, with the higher layer solving the environment arrangement problem and lower layer solving the problem of motion planning. Previously planned trajectories and past results for this design problem were first constructed as datasets; however, they cannot be seen as optimal. Therefore, this study proposed an experience-reuse manner, which selected the most “useful” experience from the datasets and reused it to query new problems, optimize the results in the datasets, and provide better environment arrangement with shorter path lengths within the same time. Therefore, a hierarchical structural caseGA-ERTC algorithm was proposed. In the higher layer, a novel approach employing the case-injected genetic algorithm (GA) was implemented to tackle optimization challenges in robot environment design, leveraging experiential insights. Performance indices in the arrangement of the robot system’s environment were determined by the robotic arm’s motion and path length calculated using an experience-driven random tree (ERT) algorithm. Moreover, the effectiveness of the proposed method is illustrated with the 12.59% decrease in path lengths by solving different settings of hard problems and 5.05% decrease in easy problems compared with other state-of-the-art methods in three small robots.

## 1 Introduction

Robotic manipulation tasks such as welding (Zeng et al., 2019), transporting (Fu et al., 2020), assembling (Tereshchuk et al., 2019), and pick-and-place are commonly utilized in intelligent manufacturing industrial robots. In these tasks, takt time (Davies, 2009), the cycle time to perform the specific operation, is seen as an important evaluation criterion for robotic manipulation to assess the efficiency of a robotic system. The longer the takt time for a task, the less efficient the robotic system becomes. In our study, to simplify the problem, we consider the length of the robotic arm’s trajectory as the moving time apart from the effect of the dynamics of the robot.

Although the primary focus of industrial robot motion planning lies in enhancing operational efficiency, it is affected by the configuration of various components within the robot environment. These components, including the base, conveyors, sensors, objects, and other robots, all play important roles during task execution, and the accuracy of the robot, which can be enhanced through calibration technology, also contributes to improving industrial automation (Li et al., 2021; Li et al., 2022a; Li et al., 2022b). Furthermore, alterations in the robotic system’s environment necessitate corresponding adjustments in the robot’s motion, even for repetitive tasks, resulting in notable variations in takt time or energy utilization (Gueta et al., 2007). Consequently, the spatial layout and arrangement of the robot environment significantly influence industrial productivity. To optimize the efficiency of a robotic system, it is imperative to establish an appropriate environment setup alongside devising efficient robot trajectories. Therefore, designing a robotic system that ensures reasonable takt time is imperative for enhancing the efficiency of industrial manufacturing.

However, most conventional studies focused on motion planning or path planning among those two aspects but ignored that with the improper design of the industrial line; the takt time could increase significantly. Hence, there are few studies that have developed the algorithm for identifying both efficient motion and environment arrangements.

Therefore, in this study, we proposed a new optimization method, caseGA-ERTC, for industrial robotic systems, which can facilitate both robot motion and environment arrangements’ optimization. Specifically, to address the difficulty of the combined optimization problem of motion planning and environment arrangement, we utilized a hierarchical algorithm and experienced reuse manner for solving our problems. The former is to decompose the complicated problems into simpler problems to reduce the calculation cost, and the latter is to reuse the past solutions in similar optimization problems to improve the results of the past solutions. Therefore, we introduced two experience-based methods for both motion planning and environment arrangement part. Based on the experiments, our proposed method can achieve the placements of conveyors in which robots could work with shorter path lengths compared with non-experience-based state-of-the-art methods.

In our previous study (Lu et al., 2023), only one robot was tested, and limited scenarios and difficulties of problems were proposed. Therefore, in this study, we aim to broaden the definition of problems and utilize information from more complicated situations and more diverse kinds of robots (in Section 4.1) to accomplish the goal of adding to the preliminary results for the comprehensive study.

The following are the contributions made to this field of study:

1. We proposed a hierarchical structure to realize the target of optimizing both environment configuration and motion planning together by decreasing the dimension of the whole environment arrangement problem;
2. We proposed building experience databases for both optimization and motion planning. Motion planning experiences gathered from simulations can also be applied to real-world robotic experiments;
3. Our proposed experience-based method could find results with shorter path lengths compared with non-experience-based methods with the same amount of optimizing time.

The remaining sections of this paper are organized as follows: Section 2 introduces related work in environment arrangement and experience-based motion planning algorithms. Section 3 provides the detailed viewpoints of the proposed methods. Section 4 explains the constructions and methods of execution of experiments. To demonstrate the advantages of our method, we compared our methods with other state-of-the-art methods solving benchmark problems in Section 5. Finally, the conclusion is described in Section 6.

## 2 Related work

### 2.1 Environment arrangement optimization problem

Most of the previous research studies focus on the adaptive motion planning algorithms to adapt to diverse and complex environments (Bulut, 2023; Jahanshahi et al., 2019; Levant and Livne, 2011), but failures are inevitable in complex environments. However, if we think about it from another perspective, if the environment itself is good enough for the robot’s motion, then it can also reduce the probability of failures and improve the efficiency of the robot’s motion planning. Therefore, the problem of environment arrangement optimization, which aims to design a suitable environment for the robot’s motions, is introduced.

In recent years, significant advancements have been achieved in the development of algorithms aimed at addressing various optimization challenges in environment arrangement. For instance, Liu et al. (2008) conducted research focusing on enhancing the efficiency and productivity of the grit-blasting process in manufacturing by employing simulation-based modeling within a robotic system.

Similarly, Gueta et al. (2007) tackled intricate optimization dilemmas related to base placement and motion planning in inspection tasks. Their approach involved employing the tabu search algorithm (Fiechter, 1994), which employs a tabu list to navigate away from local minima, to determine optimal base positions. Subsequently, basic inverse kinematics (IK) and velocity profiles were utilized in the motion planning phase to compute joint angles and angular velocities.

However, these previous studies faced challenges in addressing highly complex, high-dimensional robotic environment design problems and did not incorporate the concept of experience reuse.

### 2.2 Experience-based motion planning

The problem of motion planning in robotics has been studied for decades, and recently, learning from the past, as well as reusing and retrieving from the previous experiences, has emerged as a new trend when dealing with motion planning problems. Experience-based motion planning could reuse past path planning problem results and does not have to plan from scratch (PFS). There are mainly two targets of these experience-based methods compared with non-experience-based methods: 1) improving the quality of the planned path or 2) saving the planning time. In addition, there are mainly two approaches for demonstrating the reuse of experiences while coping with various targets: 1) reconstructing and depicting the environment variables in task-relevant areas and 2) directly reusing and exploiting previously planned motions.

There are many previous research studies to achieve the first target, reconstructing and depicting the environmental variables in task-relevant areas.

Experience graph (E-Graph) (Phillips et al., 2013), Lightning framework (Berenson et al., 2012), and its variant Thunder (Coleman et al., 2015) introduce a novel approach to motion planning by retrieving and repairing previous planned paths.

Even though the above algorithms will become more accurate and effective as the algorithm runs and the volume of database increases because of the characteristic of these algorithms to gather environmental information and reconstruct the environment, these algorithms are more useful in static environments. Therefore, if the target of the tasks or the type of robot changes, the possibility of motion planning failure increases.

To facilitate the reuse of past experiences in addressing new motion planning challenges, the integration of case-based reasoning (Abdelwahed et al., 2018) into sampling-based algorithms has been proposed. This involves storing previous experiences in a case base and selecting relevant instances for new queries based on similarity metrics.

Because of these traits, employing case-based reasoning in complex scenarios like those encountered in 6-DOF robot systems is extremely challenging.

In our research, we employed the experience-driven random tree (ERT) algorithm (Pairet et al., 2021), which directly leverages similar past experiences to enhance result quality and computation speed.

## 3 Methods

### 3.1 Problem definition

**FIGURE 1:**
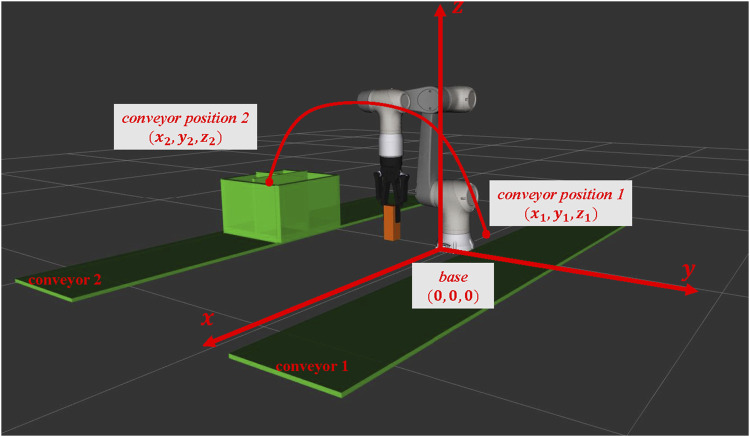
Environment settings of the problem to be solved in the 3D world coordinate. The environment is constructed with two conveyors, and one box is placed on one of those conveyors. Our task to be solved is defined as a pick-and-place task; the robot is catching one object from conveyor 1 to place it into the box on conveyor 2. However, the arrangements of the environment and the placement of the conveyors would influence the moving time for the robotic arm when executing the above tasks. To simplify the problem, we consider the length of the robotic arm’s motion trajectory as the moving time of executing the pick-and-place task. Therefore, the target of this study is to arrange the environment arrangements in specific positions of conveyors for achieving the goal of optimizing the path length of the trajectory when executing the pick-and-place tasks.

**FIGURE 2:**
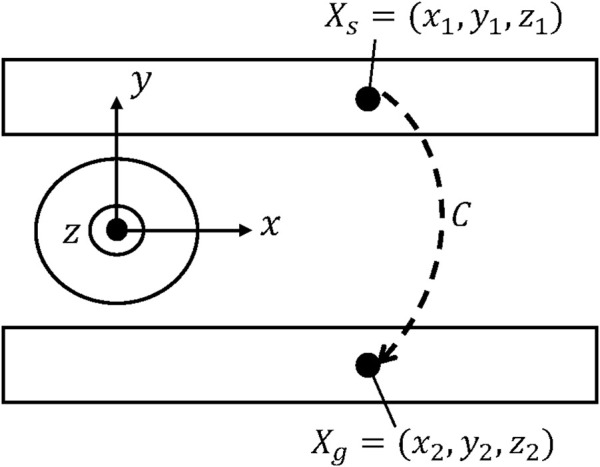
Two-dimensional environment setting of the problem to be solved (top view).

As shown in Figures 1, 2, the settings of sensors and gathering information from sensors were not considered. The relative positions of the picking object and the end-effector were fixed. Therefore, we defined the problem of environment arrangement as finding the positions of conveyors when the robot picks the object, moving from the picking C-Space $C\left(q_{\mathrm{pick}}\right)$ to a placing C-Space $C\left(q_{\mathrm{place}}\right)$ , at which the path length of the planned trajectory is the minimum.

The optimization process in question considers the path length of the motion as the primary evaluation criterion. To be precise, our objective is to determine the positions of conveyors S within the robotic environment and the associated trajectory T by the final timestep T .

Therefore, we conceptualize the task of arranging the environment as an optimization problem, where the path length l=f(T) generated by the motion planning algorithm serves as the pivotal evaluation criterion.

$$\underset{X_{S},X_{G}\in SE\left(3\right)}{\mathrm{arg min}}\;l=f\left(T\left(X_{S},X_{G}\right)\right),$$

$$\begin{array}{cc} s.t.,\;\;flag\left(T\left(X_{S},X_{G}\right)\right) & =TRUE\\ time\left(X_{S},X_{G}\right) & \leq T, \end{array}$$

where the picking configuration $C\left(q_{\mathrm{pick}}\right)$ calculated from the start Cartesian position $\left[x_{1},y_{1},z_{1}\right]$ and the orientation of the end-effector corresponding to the defined tasks. Placing configuration $C\left(q_{\mathrm{place}}\right)$ corresponds to the goal Cartesian position $\left[x_{2},y_{2},z_{2}\right]$ , and constraints are added as

$$s.t.,\;\;x_{1}=x_{2},y_{1}=-y_{2},z_{1}=z_{2}$$

to simplify the defined problem by decreasing the dimension of the environment parameters. Therefore, less computation time is required when designing the environment arrangements.

Therefore, this environment arrangement problem could be defined as a 3-dimension (x,y,z) plus a 6-dimension motion planning problem, which is a 9-dimensional optimization problem.

In addition, $flag\left(T\left(X_{S},X_{G}\right)\right)$ is a Boolean function indicating whether the robot can successfully plan the motion for the given configurations of the task. If the task is feasible, it returns true; if not, it returns false.

### 3.2 Overall structure of our proposed method

**FIGURE 3:**
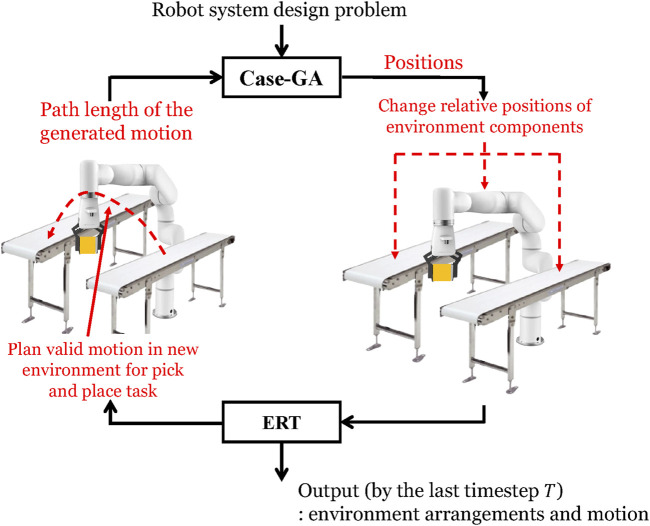
Overview of the methods.

The proposed method is structured as follows: as mentioned above, this environment arrangement problem is a high-dimensional optimization problem; therefore, the decomposition of this optimization problem is critical to solving it. This study adopts the case-GA + ERTC to solve the environment optimization problem. As shown in Figure 3, case-GA (Louis and McDonnell, 2004) is introduced first. In each generation of this genetic algorithm, the relative positions of environment components are changed to construct the new environment for the robot pick-and-place tasks.

Then, the motion planning algorithm, ERTConnect algorithm (Pairet et al., 2021), was presented here for planning valid motion in this new environment. The path length of the generated motion is returned to the case-injected GA as the evaluation criterion for the optimization process. After running the algorithm for *n* generations and by the last timestep T , the final environment arrangements and its corresponding motion when the robot executes the pick-and-place task in this environment would be output as the final results.

### 3.3 Construction of datasets

As shown in Figure 3, the problem is solved by the hierarchical construction of methods. The higher-layer optimization problem and lower-layer motion planning problem are solved. Two datasets are introduced for methods in each layer.

Casebase C , used in higher layer: case-GA, is composed of solutions. Therefore, solutions from the previously solved environment arrangement problems [x,y,z] were collected into the casebase C .

**FIGURE 5:**
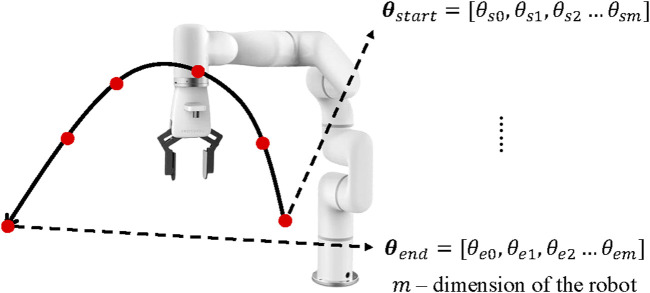
Trajectories stored in the ERTConnect algorithm dataset are time-series points in C-space.

In the lower layer, the motion planner’s dataset A was constructed with trajectory information. As shown in Figure 5, because of the discontinuity of the sampling-based methods, trajectories are composed of time-series points in C-Space. When the robot’s end-effector moves to the designated Cartesian pose $X_{G}$ , each series point provides joint angle information.

Therefore, each trajectory is demonstrated with joint angles in C-Space $\left[\theta_{0},\theta_{1},\ldots ,\theta_{n}\right]$ . The starting and ending configurations of each trajectory were saved for the comparison of similarity when selecting experiences.

### 3.4 Experience-based optimization method

Case-GA was used for the main structure of this optimization problem; therefore, the algorithm should start from the random generation of the first generation of the genetic algorithm.

For each positions of conveyors, which defines the construction of the environment, we decided for planning the motion of the robot for 10 times and chose the trajectory which has the smallest path length to mitigate the problem caused by the randomness of those sampling-based motion planning algorithms. As a result, for each individual, it will take approximately 4 s–40 s (if all failed). However, the total optimization time should be limited in real engineering industrial environment design problems.

Therefore, in this study, we set the total generation of this algorithm as 20. Here, we also defined the size of each population as 20; therefore, 20 $\left[x_{0},y_{0},z_{0}\right]$ s are generated for constructing 20 different environments. Then, the motion planning algorithm is introduced for planning the pick-and-place task in 20 previously designated environments with 20 different pick-and-place positions.

**FIGURE 4:**
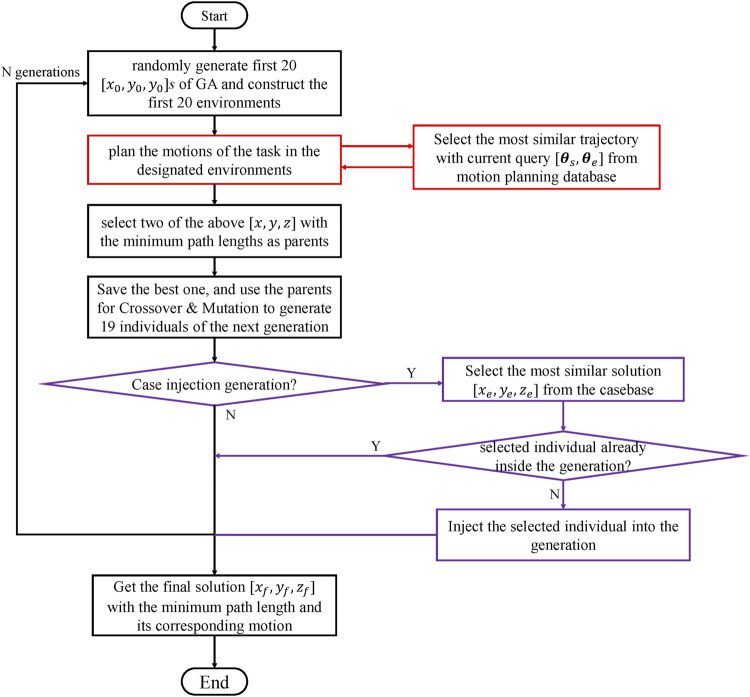
Flowchart of the proposed experience-based environment arrangement method.

The evaluation criterion revolves around the path lengths determined by the motion planning algorithm; therefore, two of the above [x,y,z] with the minimum path lengths are defined as parents. The individual with the minimum path length would be saved and directly transferred into the next generation, and two parents mentioned above would be used for generating 19 individuals for the next generation by real-coded simulated binary crossover (SBX) and polynomial mutation methods (Pattanaik et al., 2018a). Then, the case-inject process of the genetic algorithm is considered (marked as purple in Figure 4). Case injection action is conducted over a specific period of generations; here, we defined it as 4. Since the total generation of this GA was designed as 20, case injection was conducted five times in each problem of scenery.

For each case-injection action, cases with the minimum difference with the best individual in the injecting population were selected; here, we define $\left[x_{e},y_{e},z_{e}\right]$ based on the following equation:

$$a=\underset{a_{i}\in C}{\mathrm{arg min}}\;dist\left(a_{i}-d\right),$$

where d represents the best individual $\left[x_{b},y_{b},z_{b}\right]$ in the current injecting generation and $a_{i}$ represents the $\left[x_{i},y_{i},z_{i}\right]$ solutions in casebase C . When injecting cases into the population, there is a possibility that the selected case $\left[x_{e},y_{e},z_{e}\right]$ is already inside the population, especially not during the first injection. Therefore, only when the individual is not already inside the generation, the selected case would be injected. Then, the algorithm would be run for the next generation until meeting the stopping criterion. At the end of the algorithm, the final solution $\left[x_{f},y_{f},z_{f}\right]$ with the minimum path length and its corresponding motion would be returned.

### 3.5 Experience-based motion planning algorithm

Path length calculated by the motion planning algorithm in environments designated in the higher layer GA is seen as the evaluation criterion in the GA process. Therefore, experience-based planning was performed by the ERTConnect planner (Pairet et al., 2021) (marked as red in Figure 4).

Algorithm 1 ERTConnect motion planner ().

   **Input**: Positions of conveyors S ( x , y , and z ) and Motion planner’s database A.

   **Output**: Trajectory T and its corresponding path length l

1: **convert** S to start and goal configurations $q_{\mathrm{start}}$ , $q_{\mathrm{goal}}$ in C-space with the IK solver
2: select **experience** $\xi_{e}$ with the minimum Euclidean distance with the current query $q_{\mathrm{start}}$ , $q_{\mathrm{goal}}$ from the database A

3: **ERTConnect**() with experience $\xi_{e}$ plan for feasible solution T

4: **calculate**the path length l of the trajectory T

5: **end**
6: **return** l

As shown in Algorithm 1, positions of the conveyors S would be converted into start and goal configurations $q_{\mathrm{start}}$ and $q_{\mathrm{goal}}$ of the 6-DOF robot in C-Space with the IK solver since the ERTConnect motion planner searches the trajectory in configuration space.

In the selection of experiences from the database, a similarity equation is devised to assess the Euclidean distance between the configurations of the start and end states, as depicted below:

$$\xi_{e}=\underset{\xi_{e_{i}}\in A}{\mathrm{arg min}}\;dist\left(\xi_{e_{i}}\left(start\right)-q_{\mathrm{start}}\right)+dist\left(\xi_{e_{i}}\left(goal\right)-q_{\mathrm{goal}}\right),$$ (1)

where $q_{\mathrm{start}}$ and $q_{\mathrm{goal}}$ are the start and goal configurations of the current planning problem, respectively; meanwhile, ${\xi_{e}}_{i}$ is the experience path stored in library A .

Then, the ERTConnect algorithm was executed for planning trajectories connecting starting and ending configurations using the previously selected experience $\xi_{e}$ .

The fundamental operation of the ERT algorithm is similar to the Expansive Spaces Trees (EST) algorithm (Hsu et al., 1997) because of its foundation in tree-based sampling techniques. However, it diverges with its integration of the experience-reuse component, which facilitates the establishment of spatial connectivity. Additionally, the ERTConnect algorithm represents a bidirectional iteration of the ERT algorithm.

**FIGURE 6:**
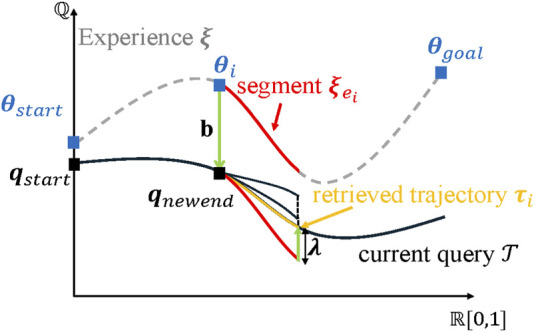
Procedure for the ERTConnect algorithm [adapted from Pairet et al. (2021)]. The experience trajectory $\xi_{e}$ is selected and set as a reference for planning a new trajectory. First, this experience trajectory $\xi_{e}$ is randomly divided into several micro-experiences (e.g., the red segment ${\xi_{e}}_{i}$ ). This experience segment will then be retrieved to a new query (in yellow) according to Equation 2 (in green). This process will be executed from the starting configuration $q_{\mathrm{start}}$ until the final configuration $q_{\mathrm{goal}}$ is realized.

During each query, the ERTConnect algorithm ascertains a seamless trajectory within the C-Space, spanning from the starting state to the target configuration, utilizing the given experience. As shown in Figure 6, the prior experience (composed of points in C-Space $\xi_{e}={\left[\theta_{0}\cdots \theta_{n}\right]}^{T}$ ) would be first randomly divided into several micro-experiences. These micro-experiences would be mapped to the current query piece by piece. For each micro-experience, the starting configuration $\theta_{i}$ would be linked to the ending configuration $q_{\mathrm{newend}}$ of the planned trajectory using affine transformation:

$$\tau_{i}=\lambda_{m\times n}\cdot {\xi_{e}}_{i}+b_{m\times 1},$$ (2)

where $b_{m\times 1}=q_{\mathrm{newend}}-\theta_{i}$ could be seen as the shifting vector used to link the starting configuration of the micro-experience and new query. In addition, m represents the dimension of the robot. $\lambda_{m\times n}=\left[0,\frac{\alpha }{n-1},\frac{2\alpha }{n-1},\ldots ,\alpha \right]$ , in which $\alpha =\frac{q_{\mathrm{goal}}-b_{m\times 1}}{\theta_{\mathrm{goal}}}$ . Therefore, the ending configuration of each micro-experience could be generated.

The path would be planned until the start and goal configurations are connected in the configuration space. In the end, the path length l of the planned trajectory T would be returned into the GA process.

## 4 Experiments

In this section, we evaluate the effectiveness of our proposed method by comparing it with other non-experience-based state-of-the-art optimization and motion planning algorithms. We assess the performance in terms of path length with different stopping criteria in simulation experiments.

### 4.1 Experimental setups

#### 4.1.1 Construction of the problems

**FIGURE 9:**
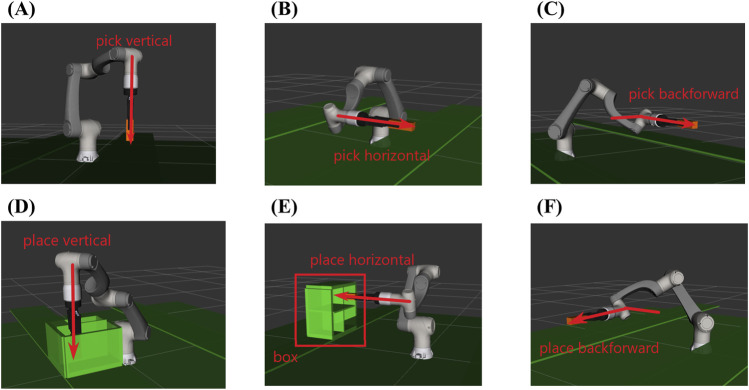
Environment constraints for defining the problem to be solved. In total, there are three picking constrains: **(A)** picking from the vertical side; **(B)** picking from the horizontal side; and **(C)** picking from the back-forward side and three placing constraints: **(D)** placing from the vertical side; **(E)** placing from the horizontal side; and **(F)** placing from the back-forward side. Whether the box [as shown in **(E, F)**] exists in the environment is also considered an environment constraint.

Regarding benchmark tests for the environment arrangement solver, we defined several environment constraints for constructing different pick-and-place tasks. As shown in Figure 9, three picking directions, namely, vertical (A), horizontal (B), and back-forward (C), and three placing directions, namely, vertical (D), horizontal (E), and back-forward (F), are designed for setting the tasks. In addition, whether the box exists in the environment is also considered an environment constraint. Therefore, in total, 36 benchmark problems are created for testing. Among them, tasks are separated into hard and easy tasks, according to the direction and existence of the box. Picking or placing from the vertical sides often involves motions that are primarily in the vertical or near-vertical plane. This can result in fewer rotational degrees of freedom required for the robotic arm. Therefore, benchmark problems when no box is in the environment or the box is placed in the vertical side are defined as easy tasks, and benchmark problems when the box placed on the horizontal side and back-forward side are considered as hard tasks. In total, 18 easy tasks and 18 hard tasks were designed.

#### 4.1.2 Implementation of the methods

For the two layers of this hierarchical structural problem-solving method, two experience-based methods and two state-of-the-art non-experience-based methods were utilized for the test. For the upper level of the hierarchical structure, which aims to solve the optimization problem, GA (Pattanaik et al., 2018b) and case-injected GA (Louis and McDonnell, 2004) are used. For the lower level of the hierarchical structure, which aims to solve the motion planning problem, motion planners RRTConnect algorithm (Kuffner and LaValle, 2000) and ERTConnect algorithm (Pairet et al., 2021) are introduced. Therefore, , in total, 2 (for higher layer) × 2 (for lower layer) = 4 combinations of environment arrangement solvers, namely, GA-RRTC, caseGA-RRTC, GA-ERTC, and caseGA-ERTC, are introduced for solving this environment arrangement problem.

#### 4.1.3 Equipment utilized for the experiments

Four robots with different sizes and configuration structures are utilized for testing the effectiveness of our proposed method:

• xArm6: 6-DOF, maximum reach: 691 mm

• Elfin5: 6-DOF, maximum reach: 800 mm

• Motoman mhj: 6-DOF, maximum reach: 909 mm

• AUBO-i7: 6-DOF, maximum reach: 1,150 mm.

#### 4.1.4 Environment settings

The center positions of the box and the left conveyor were aligned, and the sizes of the boxes and conveyors were set in proportion to the reach of each robotic arm, as stated in Section 4.1.3. The height of the box was set to 0.2R , and the width of the box was made equal to the width of the conveyor at 0.3R . The lengths of the box and the conveyor were set to 0.65R and 1.36R , respectively, where R represents the maximum reach of each robot.

### 4.2 Dataset setups

**FIGURE 7:**
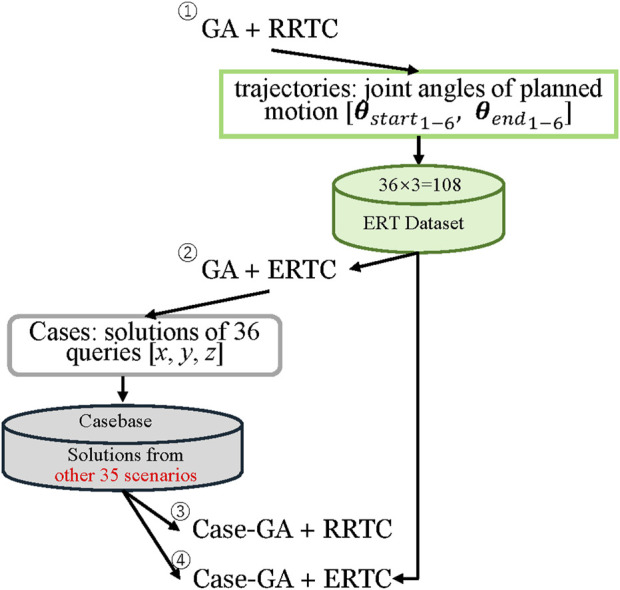
Sequence of the execution of experiments.

Dataset collection is related to the sequential execution of experiments. Therefore, we designed four steps to execute our experiments, as shown in Figure 7. In the beginning, 36 environment arrangement problems were solved via the GA-RRTC algorithm. The trajectories of the final results and other randomly selected two trajectories were stored into the dataset, which would be used for the ERTConnect algorithm in later experiments; therefore, in total, 108 trajectories were stored into the ERT dataset.

Trajectories were represented and stored by the angles of joints of the robotic arm in configuration space corresponding to each discrete point in 3D space. However, only the start configuration $\theta_{start_{1-6}}$ and goal configuration $\theta_{goal_{1-6}}$ were used in the evaluation of the similarity according to Equation 1.

Then, the same environment arrangement problems are solved by the combination of the GA-ERTC algorithm. Here, the final solutions of 36 queries ( x , y , and z ) are seen as cases to be saved into the casebase. However, to ensure the casebase used by the query does not contain the solution that was solved by the same problem, only 35 solutions would be saved into the casebase for each query. These casebases would be used for case-injected GA for the next two experiment testings: the case-injected GA-RRTC algorithm and case-injected GA-ERTC algorithm.

## 5 Results and discussion

**FIGURE 8:**
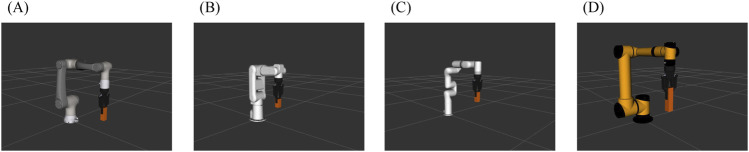
3D Model in RViz of the robots used in experiments: **(A)** Elfin5 robot. **(B)** mhj robot. **(C)** xArm6 robot. **(D)** AUBO-i7 robot.

We have done simulation experiments in four robots (AUBO-i7, Elfin5, mhj, and xArm6, as shown in Figure 8) with easy tasks and hard tasks using four combinations of methods mentioned above. Here, we access our results by calculating the average value of the path length (rad) of the 18 tasks when stopping the environment arrangement algorithm at different times. For the auboi7 robot, whose size is the largest, the time spent for planning feasible motions was longer than other robots with smaller sizes. Therefore, for the AUBO-i7 robot only, the stopping time criteria are longer than the three other robots.

**FIGURE 10:**
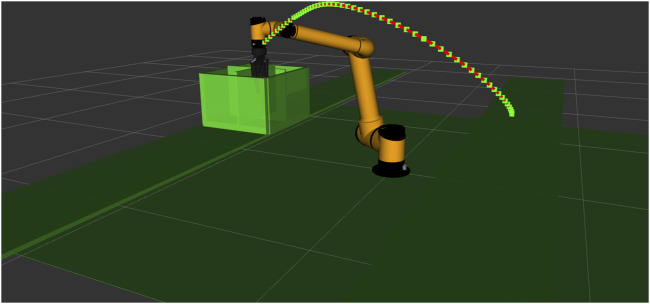
Result trajectory of the AUBO-i7 robot when performing the task involves picking from the horizontal side and placing from the vertical side.

Figure 10 shows an example of our obtained results, in which the AUBO-i7 robot was used to solve the environment optimization problem when performing the task involves picking from the horizontal side and placing from the vertical side. The final positions of the conveyors are $\left[x_{1},y_{1},z_{1}\right]=\left[0.3524,0.6594,0.3137\right]$ m and $\left[x_{2},y_{2},z_{2}\right]=\left[0.3524,-0.6594,0.3137\right]$ m, with the path length of the trajectory as 2.98 rad. According to the results obtained from the simulation shown in Table 1, the proposed experience-based environment arrangement solver case-injected GA-ERTC showed the highest performance in most of the experimental tasks, especially in robots with small sizes, such as Elfin5, xArm6, and mhj robots. When stopping the algorithm in the 2000s in easy tasks and 3000s in hard tasks, our proposed experience-based method shows better results. In experiments conducted in the Elfin5 robot, there is a decrease in the final result (path length) of 8.06% in easy tasks and 19.64% in hard tasks. In the mhj robot, there is a decrease in the final result of 0.54% in easy tasks, 8.99% in hard tasks. In the xArm6 robot, there is a decrease in the final result of 6.55% in easy tasks and 9.15% in hard tasks.

**TABLE 1:** Path length (rad) returned when stopping the optimization algorithm in different progresses in four robots (AUBO-i7, Elfin5, mhj, and xArm6).

	AUBO-i7 (easy tasks)		
Condition	Path length (1000s)	Path length (2000s)	Path length (3000s)
GA-RRT	2.407574	2.247776	2.210905
caseGA-RRT	2.515666	2.346976	2.295159
GA-ERT	2.478247	2.31395	2.259126
caseGA-ERT	2.486048	2.242487	**2.167826** (−1.9%)

	AUBO-i7 (hard tasks)		
Condition	Path length (1000s)	Path length (3000s)	Path length (5000s)
GA-RRT	2.747944	2.480159	**2.391671**
caseGA-RRT	3.041041	2.680214	2.572279
GA-ERT	2.638694	2.505068	2.469904
caseGA-ERT	2.723162	2.569482	2.459562

	Elfin5 (easy tasks)		
Condition	Path length (800s)	Path length (1400s)	Path length (2000s)
GA-RRT	1.911629	1.892187	1.880808
caseGA-RRT	1.909614	1.895111	1.886558
GA-ERT	1.838229	1.790196	1.780574
caseGA-ERT	1.787515	1.737445	**1.729197** (−8.06%)

	Elfin5 (hard tasks)		
Condition	Path length (1000s)	Path length (2000s)	Path length (3000s)
GA-RRT	3.582743	3.448506	3.378888
caseGA-RRT	3.403129	3.12497	3.073381
GA-ERT	2.985809	2.832983	2.780494
caseGA-ERT	2.956463	2.781493	**2.715406** (−19.64%)

	mhj (easy tasks)		
Condition	Path length (800s)	Path length (1400s)	Path length (2000s)
GA-RRT	2.303998	2.274186	2.236647
caseGA-RRT	2.29979	2.276456	2.259014
GA-ERT	2.254757	2.246878	2.246562
caseGA-ERT	2.234666	2.229086	**2.224507** (−0.54%)

	mhj (hard tasks)		
Condition	Path length (1000s)	Path length (2000s)	Path length (3000s)
GA-RRT	3.437869	3.323296	3.249903
caseGA-RRT	3.371937	3.235782	3.149138
GA-ERT	3.111261	3.048727	2.990963
caseGA-ERT	3.057014	2.979651	**2.960559** (−8.99%)

	xArm6 (easy tasks)		
Condition	Path length (800s)	Path length (1400s)	Path length (2000s)
GA-RRT	1.930181	1.899212	1.848715
caseGA-RRT	1.939437	1.911066	1.866544
GA-ERT	1.794222	1.784214	**1.775055**
caseGA-ERT	1.835677	1.811967	1.793110 (−6.55%)

	xArm6 (hard tasks)		
Condition	Path length (1000s)	Path length (2000s)	Path length (3000s)
GA-RRT	3.383504	3.259521	3.158789
caseGA-RRT	3.78115	3.415445	3.380669
GA-ERT	3.209672	2.973886	2.939468
caseGA-ERT	3.130803	2.976547	**2.869729** (−9.15%)

The best results are highlighted in bold

However, when stopping the algorithm in 5000s in hard tasks of the large robot AUBO-i7, our proposed experience-based method did not show good performance. There might be mainly two reasons for this phenomenon.

1) The experience-reuse efficiency in the AUBO-i7 robot is lower than other robots. As shown in Table 2, we calculated the average distance (rad) between the selected experience and current query when using the ERTConnect algorithm and compared the results between the AUBO-i7 robot and Elfin5 robot. The results show that the distance in AUBO-i7 cases is approximately twice that of Elfin5 cases. This represents that experiences reused in AUBO-i7 could be more different with a new query; therefore, the experience-reuse efficiency would be lower than small robots.
2) The hard tasks of the AUBO-i7 robot are more difficult than other robots because of the environment settings (the set of the box). We set the size of the boxes in proportion to the reach of the robots, not scaled to the robot’s configuration space. Thus, the large size of the robot AUBO-i7 increases the potential for collisions. Therefore, the motion planning in the large robot, AUBO-i7, is much harder than that in small robots, which leads to the increase in final results (path length) in hard tasks of the AUBO-i7 robot.

**TABLE 2:** Average distance (Rad) between the current query and selected experience of AUBO-i7 and Elfin5 robot in the pick-and-place task from the back-forward side when there is box in the environment.

	AUBO-i7	Elfin5
Average distance (rad)	3.8717168	1.2448932

In reason 1, average distances between current query and selected experiences in the AUBO-i7 robot are higher than those in the Elfin5 robot. The reason for this might be related to the second reason. When the positions of the boxes change in the environment, because of the high difficulty of planning feasible motions of the AUBO-i7 robot, even the small changes in the positions of the box could lead to a large slip of the starting and ending configurations compared with the dataset planned by the IK module at the first stage of motion planning.

In addition to the abovementioned results, we also introduce a laddered reference for users who are willing to use our proposed methods. In real manufacturing, tolerable planning time might be shorter than our proposed maximum stopping time. Therefore, our results also show how long the path lengths of the designed environments can be in a shorter planning time (800s, 1400s, and 2000s), which could be reused in practical environment arrangement problems for engineers.

## 6 Conclusion and future work

In this study, we proposed an experience-based environment arrangement solver to solve the combination problem of environment arrangement and motion planning. To solve this high-dimensional problem, the hierarchical structure is proposed to decompose the high-dimensional problem into two semi-high-dimensional problems. Case-injected GA was used for solving the optimization problem, and the path length planned by the ERTConnect algorithm was used as an evaluation criterion in the process of genetic algorithm. We compared our proposed experience-based methods with non-experience-based state-of-the-art methods.

The results of simulation experiments showed that our proposed experience-based method case-injected GA-ERTC algorithm can sufficiently re-utilize the past experiences and design the environment with the average 5.05% decrease in path length in 18 easy and 12.59% in 18 hard pick-and-place tasks in three robots (Elfin5, mhj, and xArm6).

However, there are still several limitations in this study. First, as shown in our results, the effectiveness of our proposed method could be unclear when being used in the large robot while increasing the difficulty of tasks. Second, our goal is to solve problems and increase the quality of the final results within specified limited time, so we do not seek to ensure the optimality of the result. Third, we have only tested our proposed method on 6-DOF robots, so it would be another interesting study if we could extend our vision to 7-DOF robots.

Therefore, there could be three future directions for our work. First, for different types of tasks, a switch between methods can be proposed. For simpler tasks without obstacles, non-experience-based methods, which do not require referencing previous experiences, could be employed to increase the calculation speed. However, for more complex tasks where the success rate of non-experience-based motion planning methods is low, experience-based approaches would be more suitable. Second, discussion about the optimality of the optimization algorithms could be another direction. Third, since our proposed methods are only tested on 6-DOF robots, the transfer of experiences among 6-DOF robots and 7-DOF robots could also be an interesting research topic.

## Data Availability

The raw data supporting the conclusions of this article will be made available by the authors, without undue reservation.
